# Altered Functional Connectivity in Children with ADHD Revealed by Scalp EEG: An ERP Study

**DOI:** 10.1155/2021/6615384

**Published:** 2021-05-11

**Authors:** Chunli Chen, Huan Yang, Yasong Du, Guangzhi Zhai, Hesheng Xiong, Dezhong Yao, Peng Xu, Jianhua Gong, Gang Yin, Fali Li

**Affiliations:** ^1^School of Life Science and Technology, Center for Information in Medicine, University of Electronic Science and Technology of China, Chengdu 611731, China; ^2^China National Clinical Research Center on Mental Disorders (Xiangya), Changsha 410011, China; ^3^China National Technology Institute on Mental Disorders, Changsha 410011, China; ^4^Hunan Technology Institute of Psychiatry, Changsha 410011, China; ^5^Hunan Key Laboratory of Psychiatry and Mental Health, Changsha 410011, China; ^6^Mental Health Institute of Central South University, Changsha 410011, China; ^7^Mental Health Center Affiliated to Medical School of Shanghai Jiao Tong University, 200030, China; ^8^Shenzhen Nao Qianneng Co., Ltd., 518002, China; ^9^Luohu District Maternity and Child Healthcare Hospital, Shenzhen 518019, China; ^10^Sichuan Cancer Hospital, School of Medicine, University of Electronic Science and Technology of China, Chengdu 610041, China

## Abstract

Attention deficit hyperactivity disorder (ADHD) is one of the most common neurodevelopmental brain disorders in childhood. Despite extensive researches, the neurobiological mechanism underlying ADHD is still left unveiled. Since the deficit functions, such as attention, have been demonstrated in ADHD, in our present study, based on the oddball P3 task, the corresponding electroencephalogram (EEG) of both healthy controls (HCs) and ADHD children was first collected. And we then not only focused on the event-related potential (ERP) evoked during tasks but also investigated related brain networks. Although an insignificant difference in behavior was found between the HCs and ADHD children, significant electrophysiological differences were found in both ERPs and brain networks. In detail, the dysfunctional attention occurred during the early stage of the designed task; as compared to HCs, the reduced P2 and N2 amplitudes in ADHD children were found, and the atypical information interaction might further underpin such a deficit. On the one hand, when investigating the cortical activity, HCs recruited much stronger brain activity mainly in the temporal and frontal regions, compared to ADHD children; on the other hand, the brain network showed atypical enhanced long-range connectivity between the frontal and occipital lobes but attenuated connectivity among frontal, parietal, and temporal lobes in ADHD children. We hope that the findings in this study may be instructive for the understanding of cognitive processing in children with ADHD.

## 1. Introduction

Attention deficit hyperactivity disorder (ADHD) is one of the most common childhood psychiatric disorders, characterized by age-inappropriate symptoms of inattention, hyperactivity, and impulsivity [1]. It arises in childhood and often persists into adolescence, even adulthood. The reduced-capacity [2] or dysfunctional attention [3] was not the major cause of ADHD symptoms; event-related potential (ERP), such as P2, N2, and P3, has consistently suggested the cognitive deficits in multiple stages of sensory and cognitive processing in ADHD [4]. P2, a positive deflection peaking around 150-250 ms after stimuli presentation, plays a crucial role when measuring attention [5], which could reflect not only early comprehension but also index updating of a representation in response to new incoming information [6]. A smaller P2 in ADHD reflects an underactivation of the early orienting process and a poor allocation of attentional resources [7]. Besides, N2, peaking at around 200-250 ms after stimuli onset, could reflect the cognitive control for successful inhibitory control and interference suppression [8] and has also been found to be reduced in ADHD patients [9]. P3 is a later ERP component and peaks around 250-600 ms and is widely used to investigate cognitive mechanisms in neuropsychiatric disorders, including ADHD [10]. For example, Li et al. suggested that the rest and task P3 electroencephalogram (EEG) could actually provide comprehensive information to reliably classify schizophrenia patients from healthy controls (HCs) [11]. Additionally, its amplitude has also been found to be decreased in ADHD [12]. As illustrated, the early stage may influence subsequent response processes [13]; since P2, N2, and P3 index different stages of cognitive information processing, in this work, all of these components were investigated to uncover the cognitive deficits in ADHD children.

The brain is not only a dynamic system but also a complex network [14]. Of note, other than isolated brain regions, the brain networks could reveal the information exchange and propagation among various brain regions [15] and provide more information to shed light on the relationship between cognition and the related brain network [16, 17]. Deficits of certain regions disturb the information processing in the brain and consequently lead to network failure [18]. ADHD was described as a network disorder [19], as ADHD has been demonstrated to be linked not only with the structural deficits of specific regions but also with the structural interconnectivity [20]. Structural magnetic resonance imaging studies have reported abnormal volume and cortical thickness in multiple brain regions [21], including prefrontal [22], temporal, and parietal [23] cortices, in ADHD. Network neuroscience [24] thus raised a “bridge” between ADHD and brain network analysis [25]. For example, Furlong et al. found that the increased global network efficiency is associated with elevated ADHD symptom severity [26]. Srinivasan suggested a static state of deficient connectivity in ADHD and a stimulus-induced state of overconnectivity within and between frontal hemispheres [27]. To understand the network mechanism underlying ADHD, we subsequently utilized the brain network analysis to probe the stimulus-induced network structure of ADHD children.

In this study, we hypothesized that the differences in ERP components could reflect on which stage the cognitive deficits in ADHD occurred, and brain network analysis might further uncover the potential network mechanism underlying the task behaviors of ADHD during the designed P3 tasks. Therefore, to better understand how ADHD children recruit cognitive resources to complete the tasks, the ERP and complex network analyses were adopted to investigate the potential differences between HCs and ADHD children.

## 2. Materials and Methods

### 2.1. Participants

A total of 71 children, including 40 ADHD children (35 males; mean age = 7.65 years, standard deviation (SD) = 2.11 years) and 31 HCs (20 males; mean age = 7.68 years, SD = 2.36 years) were recruited from Shenzhen Luohu District Maternity and Child Healthcare Hospital, China. ADHD children needed to meet the *Diagnostic and Statistical Manual of Mental Disorders* fifth edition (DSM-5) criteria of ADHD diagnosis. Two psychiatrists made these diagnoses independently based on DSM-5 and needed to assess the children in two or more situations. For the control group, age-matched children did not have ADHD or any other psychiatric disorders. Exclusion criteria were neurological disorder, a history of brain injury, mental retardation, comorbid current psychiatric diagnosis, and other neurodevelopmental disorders.

### 2.2. Experimental Procedures

**Figure 1:**
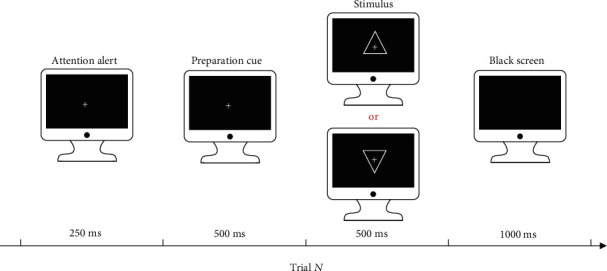
Experimental protocol of the visual P3 tasks used in this study. In each trial, a 250 ms attention alert, a 500 ms preparation cue, a 500 ms stimulus presentation, and a 1000 ms break were included. The upward-oriented triangles and down-oriented triangles with a thin cross in the center denoted standard and target stimuli, respectively. Only standard or target stimulus appeared once in one trial.

Our experiment complied with a standard oddball paradigm, as shown in Figure 1. The P3 task designed contained 30 target trials that needed the corresponding responses from subjects, along with 120 standard stimuli that did not require the subjects' responses, and during the whole P3 task, the stimuli were randomly presented. Herein, the standard stimulus was defined as the upward-oriented triangle with a thin cross in its center, while the target stimulus was defined as the down-oriented triangle with a thin cross in its center. In each trial, a 250 ms attention alert, a 500 ms preparation cue, a 500 ms stimulus presentation, and a 1000 ms break were included. Subjects were required to press the “1” key on a standard keyboard as quickly as possible once the target stimulus was presented.

### 2.3. EEG Data Acquisition

The EEG data were recorded with a 16-channel Ag/AgCl (i.e., Fp1/2, F3/4, C3/4, P3/4, O1/2, F7/8, T3/4, and T5/6) electrode cap (BrainMaster, Inc., Shenzhen, China). All electrodes were positioned according to the 10-20 international system. During online data recording, electrode AFz served as the reference. EEG signals were sampled at 1000 Hz and online bandpass filtered at 0.05-100 Hz. During recording, the impedances of all electrodes were kept below 5 k*Ω*.

### 2.4. Behavioral Data Analysis

Reaction accuracy (RA) was the ratio of the number of correct responses to the total number of stimuli. Reaction time (RT) was measured from the onset of the stimulus to the time for response (pressing the “1” key) as indicated in the recordings. Any potential differences in RA and RT between the HCs and ADHD children were investigated by an independent *t*-test.

### 2.5. EEG Data Analysis

#### 2.5.1. EEG Data Preprocessing

Multiple preprocessing procedures, including References Electrode Standardization Technique (REST) referencing [28–29], [1, 30] Hz bandpass filtering, 1-s-length segmenting (200 ms before and 800 ms after targets onset, [-200, 800] ms) (0 ms denotes stimuli onset), [-200, 0] ms baseline correction, and artifact-trial removal using a threshold of ±100 *μ*V were implemented to preprocess the EEG datasets. In fact, to evoke the clear P3, subjects have to pay all of their attention to the target stimuli, by mentally counting the target number or physically pressing the required button [30], which was clarified as the maintained attention to the targets rather than the essence of P3 generation [31]. After carefully checking the evoked ERPs of these left trials, the remaining 25.45 ± 2.78 and 26.84 ± 1.75 trials for HCs and ADHD children were included in the following analysis, respectively, which was consistently larger than the minimum trial number (i.e., 20) suggested previously [32].

#### 2.5.2. ERP Components

After preprocessing, all artifact-free trials were trial-averaged for each subject. Then, according to previous studies [7, 33], the relatively broad intervals of [150, 200] ms for P2, [200, 250] ms for N2, and [250, 600] ms for P3 were applied to the averaged ERP, to extract the corresponding P2, N2, and P3 amplitudes. Herein, the corresponding amplitude per ERP component was calculated as the mean amplitude in a 20 ms window centered at its peak. Thereafter, the differences in P2, N2, and P3 between the HCs and ADHD children were statistically investigated by an independent *t*-test.

#### 2.5.3. Brain Network

The phase lock value (PLV) was proposed to estimate the phase synchronization among each pair of signals [34] and has been widely used in neuroscience research, such as ADHD [35]. The corresponding PLV value is in a range of [0, 1] with higher values representing the stronger rhythm locking.

To estimate the corresponding instantaneous phases, *ϕ*_*x*_(*t*) and *ϕ*_*y*_(*t*), of two given signals, *x*(*t*) and *y*(*t*), the analytical signal *H*(*t*) is defined by the Hilbert transform (HT) as follows:

(1) $$\begin{array}{c} \left\{\begin{array}{c} H_{x}\left(t\right)=x\left(t\right)+i\hat{x}\left(t\right),\\ H_{y}\left(t\right)=y\left(t\right)+i\hat{y}\left(t\right), \end{array}\right. \end{array}$$

where $\hat{x}$(*t*) and $\hat{y}$(*t*) are the HT of *x*(*t*) and *y*(*t*), which are defined as follows:

(2) $$\begin{array}{c} \left\{\begin{array}{c} \hat{x}\left(t\right)=\frac{1}{\pi }\text{P}.\text{V}.\int_{-\infty }^{\infty }\frac{x\left(\tau \right)}{t-\tau }d\tau ,\\ \hat{y}\left(t\right)=\frac{1}{\pi }\text{P}.\text{V}.\int_{-\infty }^{\infty }\frac{y\left(\tau \right)}{t-\tau }d\tau , \end{array}\right. \end{array}$$

where P.V. stands for the Cauchy principal value. Then, the corresponding instantaneous phases, *ϕ*_*x*_(*t*) and *ϕ*_*y*_(*t*), can be computed as follows:

(3) $$\begin{array}{c} \left\{\begin{array}{c} \phi_{x}\left(t\right)=\arctan\frac{\hat{x}\left(t\right)}{x\left(t\right)},\\ \phi_{y}\left(t\right)=\arctan\frac{\hat{y}\left(t\right)}{y\left(t\right)}. \end{array}\right. \end{array}$$

Finally, the PLV value can be estimated as follows:

(4) $$\begin{array}{c} w^{\text{plv}}=\frac{1}{N}\left|\overset{N-1}{\underset{n=0}{\sum }}e^{i\left(\phi_{x}\left(n\Delta t\right)-\phi_{y}\left(n\Delta t\right)\right)}\right|, \end{array}$$

where *w*^plv^ is the connection weight estimated by PLV, Δ*t* is the sampling interval, and *N* denotes the sample number.

Based on the PLV, the adjacency matrix per trial per subject was first calculated. Afterwards, for each subject, the final weighted network, a 16 × 16 adjacency matrix, was acquired by averaging matrices across all trials. Based on these constructed brain networks, an independent *t*-test was used to investigate the potential difference in brain architecture between the two groups.

#### 2.5.4. Source Localization

In our present study, when estimating the cortical current density of P2 and N2, the sLORETA (v20171101) was adopted. The cortex has been modeled as a collection of volume elements (voxels) in the digitized atlas provided by the Brain Imaging Center, Montreal Neurological Institute (MNI) [36]. The sLORETA method found a particular solution to the nonunique EEG inverse problem by assuming similar activation of neighbouring neuronal sources, followed by an appropriate standardization of the current density, producing images of electric neuronal activity [37]. Regarding the technical details of the sLORETA procedure, the MNI brain volume was scanned at a resolution of 5 mm. Voxels were retained when they were unambiguously labeled as cortical gray matter and when they were unambiguously within the brain compartment. sLORETA's solution space was therefore restricted to that cortical and hippocampal gray matter whose images represented the power in 6239 voxels, with a spatial resolution of 5 mm. Anatomical labels were reported using an appropriate correction from MNI to Talairach space [38]. Besides, the lead field matrix was computed via a standardized boundary element method model volume conductor model with the realistic head model [39]. Herein, based on sLORETA, we estimated the current density of each ERP component for both HCs and ADHD children. The mean of sLORETA solutions corresponding to the ERP components was obtained within their time interval within which the ERP components were extracted. Then, the differences corresponding to the cortical activity between the two groups were statistically investigated by an independent *t*-test.

## 3. Results

### 3.1. Behavior

No significant differences in RA and RT between the two groups were first found in this study, as shown in Table 1.

### 3.2. ERP

**Figure 2:**
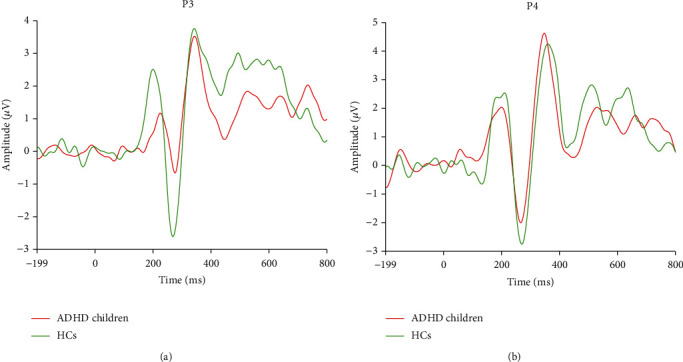
ERP waveforms at electrodes P3 (a) and P4 (b) between the HCs and ADHD children.

Thereafter, when investigating the concerned ERP components on electrodes P3 and P4, similar results on both electrodes were found. In detail, no significant difference in P3 was found between the HCs and ADHD children. On the contrary, the amplitudes of both P2 and N2 were reduced in ADHD children, when compared to HCs (*p* < 0.05), as displayed in Figure 2.

**Figure 3:**
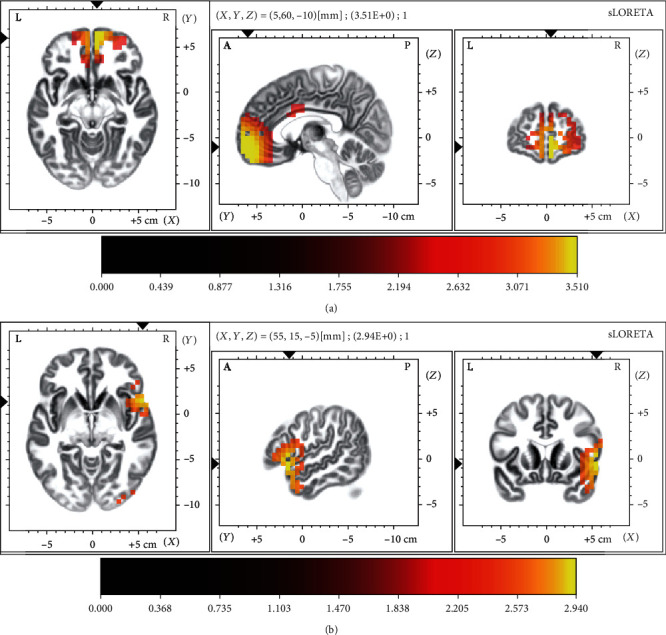
The *T*-value statistical map corresponding to the P2 and N2. (a) The stronger activity for P2 in HCs; (b) the stronger activity for N2 in HCs. The distinct colors presented the activity of the related brain regions in each subfigure.

Given the differences in N2 and P2, the differences corresponding to the cortical activity between the two groups were statistically investigated, which are shown in Figure 3. In detail, compared to ADHD children, HCs showed much stronger activity in frontal, cingulate, and central areas for P2 (*p* < 0.01) and in temporal, frontal, and occipital regions for N2 (*p* < 0.01), whose MNI coordinates, *T*-value, and voxel number are listed in Tables 2 and 3, respectively.

### 3.3. Brain Network

**Figure 4:**
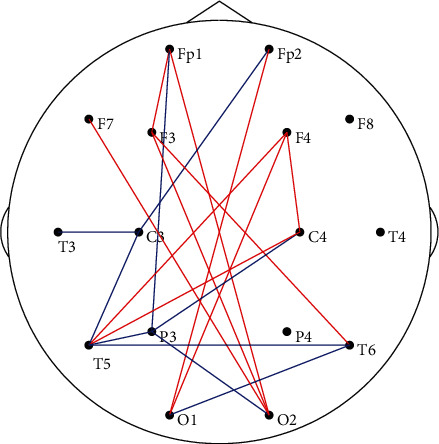
Differentiated brain topology between the HCs and ADHD children; the red and blue solid lines denote the stronger and weaker functional connectivity in ADHD children, respectively, when compared to HCs.

Figure 4 demonstrates the identified topological differences (*p* < 0.05) between the two groups. In detail, relatively stronger functional connectivity (i.e., red long-range edges) among frontal and occipital lobes but weaker linkages (i.e., blue edges) among frontal, parietal, and temporal regions were found for ADHD children, when compared to HCs.

## 4. Discussion

As previously illustrated, ADHD patients usually showed the task deficits in varied substage information processing, such as information integration and neuronal response stages, rather than the whole task period [5]. However, task behaviors usually measured the overall performance but not substage information processing [40], as behaviors were insensitive to detect the substage performance; by contrast, related ERP analysis might be helpful. Given the insignificant difference in behavior between the HCs and ADHD children, in this study, we further explored any potential difference in electrophysiological ERP and functional network to clarify underlying cognitive deficits in ADHD children.

Being exposed to external stimuli, to achieve satisfying task performance, the brain has to receive, integrate, process, and respond to their perceived target stimuli. P3 is the neuronal response to target stimuli after the decision process [41]. Li et al. [42] found that the central area served as the focal source to regulate whole-brain activities by sending out commands during the decision process, which ended when the P3 peak occurred. When investigating the underlying basis by the ERP, first at the response stage, our current study did not find a significant difference in P3 between the HCs and ADHD children, and this might indicate that the cognitive deficits between the two groups might not occur in the response stage but other ones, such as the information integration stage, which could be identified by the other components, like P2 and N2. In fact, in our present study, the corresponding electrophysiological differences in both P2 and N2 did exist between the two groups.

P2 is regarded as the exogenous response, as it is automatically produced regardless of the task or attention variables but is amenable to attentive manipulations, and its latency and amplitude may covary with aspects of selective attention or stimulus encoding processes [43]. Therefore, P2 involves in the early sensory stages of target detection [44], encoding, and classification [45]; a smaller P2 suggests an underactivation of the early orienting process and a poor allocation of the attentional resources in ADHD [46]. In this study, consistent with the previous study [47], ADHD children were found to have reduced P2 amplitude, which may be due to the lack of early attention to the stimuli. Moreover, N2 has been demonstrated to relate to the initial stimulus categorization in the selective attention stream [48] and was specific to the visual modality, which might reflect the degree of attention required for processing stimuli in the visual cortex [49]. Perhaps due to the lack of reasonable allocation of visual resources, attenuated N2 in ADHD children was found in this study. Altogether, these findings indicated that the differences in the cognitive process between the HCs and ADHD children may indeed lie in the early stage (i.e., information integration), rather than the later one (i.e., neuronal response). Therefore, ADHD children showed deficiency in automatization of the initial stimulus categorization for which they compensated by later controlled attention processes and information processing, which was earlier proposed by Karayanidis et al. [50].

More evidence underlying these differences would be acquired, when considering the cortical N2 and P2 activity. On the one hand, compared to ADHD children, HCs showed much stronger activity in frontal, cingulate, and central areas for P2. And these cortexes involved in the generation of P2 [51] and abnormalities in these regions were common in ADHD [52]. On the other hand, the temporal, especially superior temporal cortex, and frontal regions illustrated by the earlier study were both linked with N2 generators [53, 54]; in specific, the neuronal activity of frontotemporal regions was associated with N2 [55]. The reduced activity of the right temporal lobe in this study was consistent with that reported by Rubia et al. who found reduced activation in the right superior temporal lobe in ADHD patients during their attention tasks [56]. Besides, the occipital cortex contributes to attention processing [57] and has also been reported to be abnormal in ADHD [58].

Figure 4 further displays the atypical interactions underlying the task information processing for ADHD in which long-range connectivity between the frontal and occipital lobes could be found. In fact, during the visual task, the occipital lobe is responsible for receiving and integrating visual information, which was revealed by Li et al.'s study on constructing large-scale cortical networks for P3 [59], while the frontal lobe contributes to a wide range of cognitive functions, such as attention [60], decision-making [61], and executive control [62]. The frequency-specified synchronization between these regions can effectively modulate cognitive information processing [63]. And studies of both rats and humans have further consistently proved that long-range linkages between the frontal and occipital regions facilitate stronger visual evoked potentials [64, 65]. In addition, Li et al. found that the long-range frontal-occipital connectivity played crucial roles in P3 generation [66]. The enhanced frontal-occipital connectivity may alternatively compensate for the deficit in the early information integration stage, which thus facilitated their accomplishing the P3 task.

Given that only EEG datasets of several ADHD girls were collected, one possible limitation was that our present study did not take the gender effect into consideration; in the future, the gender-matched ADHD children will be recruited to validate if the gender could affect our present findings. Another might be that the task behaviors were not taken into consideration when selecting reliable task trials; in our future, more detailed individual behaviors would be recorded and then analyzed during preprocessing to further validate our current findings.

## 5. Conclusion

Although no significant differences in task behavior between the HCs and ADHD children were found, electrophysiological ERP and functional network did uncover the potential cognitive deficits in ADHD children, especially in the early task stage. In particular, significant differences in both N2 and P2 amplitudes and cortical activity, but not in P3, between the two groups were first found. And further, the topological differences showed the attenuated functional connectivity among frontal, parietal, and temporal lobes in ADHD children, which might be compensated by the enhanced long-range frontal-occipital connectivity to accomplish the required tasks.

## Figures and Tables

**Table 1:** Behavioral data.

	HCs		ADHD children		*p* value
	Mean	SD	Mean	SD	
RA (%)	65.7	21.1	58.3	22.7	*p* > 0.05
RT (s)	651.5	153.3	648.9	158.4	*p* > 0.05

**Table 2:** The details of the stronger activity for P2 in HCs.

Activated region	L/R	BA	MNI coordinates			*T*-value	Numbers of voxels
			*x*	*y*	*z*		
Medial frontal gyrus	L/R	9,10,11,25	5	60	-10	3.51	111
Superior frontal gyrus	L/R	10, 11	5	60	-20	3.51	92
Anterior cingulate	L/R	10,24,32,33	5	55	0	3.45	60
Postcentral gyrus	L/R	1, 2, 3, 40	-50	-20	60	2.49	30
Middle frontal gyrus	L/R	10, 11	25	55	-10	2.78	22

**Table 3:** The details of the stronger activity for N2 in HCs.

Activated region	L/R	BA	MNI coordinates			*T*-value	Numbers of voxels
			*x*	*y*	*z*		
Superior temporal gyrus	R	21,22,38	55	15	-5	2.94	58
Inferior frontal gyrus	R	13,44,45,47	55	15	5	2.89	58
Middle temporal gyrus	R	19,21,38	55	10	-25	2.79	36
Middle occipital gyrus	R	18, 19	40	-90	5	2.53	25

## Data Availability

The data used to support the findings of this study are available from the corresponding authors upon request.
